# Supplementary material for: Effect of virtual interactions through avatar agents on the joint Simon effect
Source: PLoS One. 2025 Jan 10;20(1):e0317091. doi: 10.1371/journal.pone.0317091 (PMC11723602; doi:10.1371/journal.pone.0317091)
Supplement: Attachment — Submitted filename: Response_to_Reviewers.docx [file pone.0317091.s006.docx]

Response to the review of “Effect of Virtual Interactions Through Avatar Agents on the Joint Simon Effect” by Y. Harada, T. Arima, & M. Okada

Thank you very much for your helpful review of the manuscript submitted to *PLOS ONE*. Based on the two reviewers’ comments and suggestions, we have made some changes to the manuscript. Comments provided by the two reviewers (in italics) and our specific responses to them are shown below. The page and line numbers in our responses indicate those used in the revised manuscript.

**Reviewer: #1**

**Comment #1.1**

*The authors specifically investigate the role of gaze visualization on the joint simon effect. The introduction could benefit from citing studies on the role of avatar’s gaze visualization, compared to human gaze, in general, not specifically to the simon effect. Also, the authors should mention a recently publish article on the effect of co-avatar visual representation on the joint simon effect (*[*https://doi.org/10.1145/3652920.365293*](https://doi.org/10.1145/3652920.365293)*)*

**Response**: Thank you for sharing the details of previous studies. Following this constructive comment, we have taken two actions. First, we cited a previous study that investigated the effect of an avatar’s gaze on social communication (p. 8, lines 110–111). The manuscript reads: “Gaze visualization has been reported to facilitate social interactions even when the gaze is produced by avatars [36].” Second, we cited Li et al. (2024) to discuss the effect of virtual interaction on the joint Simon effect (p. 7, lines 92–96). The manuscript reads: “Recently, Li et al. [27] investigated the effect of virtual interactions using avatars on the joint Simon effect. The results showed that the virtual interaction produced a statistically reliable joint Simon effect, and that the strength of the effect was weakened when the hands of the avatar were visible, but the body was not.”

**Comment #1.2**

*At page 10 the authors mention that “participants were positioned at the left or right side of the environment”. However, how it was chosen, for each participant to be placed to the left or right of the environment is not specified. Was this factors counterbalanced across participants? Moreover the procedure is not clear. How were participants asked to deliver responses? It is mentioned: “touch a corresponding button”. However, it is not clear to me whether they had to just press a button on the controllers participants held in their hands or on the display?*

**Response**: Thank you for your comment. To clarify the details of the experimental procedure, we have added two descriptions as follows: (a) “The lateral positions were counterbalanced across the participants.” (p. 11, lines 165–166); (b) “The participants’ task was to touch the corresponding button that was virtually set in front of them when the target color was presented. This touch was performed as accurately and quickly as possible, and collisions between the avatar’s hand and the button were detected. No feedback was provided for the correctness of responses.” (p. 12, lines 178–181). Moreover, we uploaded Supporting Videos 1 and 2 to clarify the trial sequence.

**Comment #1.3**

*In experiment 2, procedure paragraph, the authors mention that the order of experimental conditions was “almost” counterbalanced across participants. Why was the order not perfectly counterbalanced?*

**Response**: Thank you for your comment. This is because a reschedule of order was required by the system trouble of gaze visualization. To explain this, relevant sentences have been revised as follows (p. 17, line 270–274): “The order could be not fully counterbalanced because a reschedule of order was required by the system trouble of gaze visualization. To evaluate the balance of condition order, we calculated the frequency for each of the four joint conditions and order. A chi-square test showed that the order was not significantly unbalanced across the four joint conditions [*χ*^2^ (9) = 1.455, *p* = .997]. This indicates an almost counterbalanced order.”

**Comment #1.4**

*For both experiments, did participants have a maximum amount of time to respond? Was feedback provided for errors? In the instructions, was speed stressed or accuracy? Not mentioned.*

**Response**: Thank you for your insightful comment. To answer these questions, we have revised and added sentences as follows: “Subsequently, a target square was presented at the left or right side of the display until a response or 2 seconds elapsed.” (p. 12, lines 177–178); “No feedback was provided for the correctness of responses.” (p. 12, line 181); “For the button touch, participants were instructed to give priority to accuracy followed by speed.” (p. 12, lines 174–175); and “The trial sequence was the same as that in Experiment 1 except for some instructions. In Experiment 2, participants were instructed to give priority to speed followed by accuracy. This is because requiring quick responses would sharpen the effect of gaze visualization on the RTs.” (p. 17, lines 266–269).”

**Comment #1.5**

*Parametric tests were adopted. However, it is not clear whether data was distributed normally. Even for RTs, given the little amount of data per participants, the use of parametric tests can be problematic.*

**Response**: We agree that the repetitions in our experiments were smaller than those in previous studies. However, we are concerned that the significance tests on medians may lead to difficulty in comparing the present and previous results that analyzed mean. To adjust the distribution of response times, several studies have conducted significance tests on log-transformed response times. Therefore, in the revised manuscript, we analyzed the means of log-transformed response times. Based on these, we have added sentences as follows (pp. 13–14, lines 206–209): “Moreover, the repetitions of trials in each condition were smaller than those in previous studies [7,18], which can bias the distribution of RTs. To adjust the distribution, we conducted statistical significance tests on the means of log-transformed RTs as in several previous studies that analyzed RTs [40,41].” Based on the current analyses, we have revised statistical values in the Results and Discussion section.

**Comment #1.6**

*Also, at page 16 the authors mention the use of a 2-way ANOVA for reaction times. Why was a repeated-measures 2 way ANOVA not used, instead, as in the subsequent analysis.*

**Response**: The description in the previous draft was inaccurate. Indeed, we used repeated-measures two-way ANOVAs to analyze data in both experiments. We have revised the sentence as follows (p. 18, lines 287–288): “A repeated-measures two-way ANOVA was conducted on the log-transformed RTs with factors of five conditions and compatibility.”

**Comment #1.7**

*Following the significant main effect of condition on reaction times, the authors further explore the effect of gaze on RTs. They specifically compare the effect of partner-gaze vs. own-gaze visualization. However, this choice is not motivated. It remains unclear why the authors did not include all gaze conditions in this analysis. Specifically, it would have been more interesting to compare partner vs. both gaze visualization. In fact, in real life, people almost never perceive their gaze only.*

**Response**: Thank you for your comment. We already compared the effects of own and partner’s gaze visualization in the previous manuscript, but the description was not salient. Therefore, we have revised the description as follows (p. 19, lines 302–306): “Experiment 2 manipulated the visualizations of own and partner’s gaze individually. To compare the effects of own and partner’s gaze visualization, we also conducted a two-way repeated measures ANOVA on the joint Simon effect with factors of own and partner’s gaze (absent and present in each factor). The result showed that neither any main effect nor interaction was significant (*F*s < 0.305, *p*s > .583, *ηp*^2^s <.007).”

**Comment #1.8**

*The results of experiment 1 are of little use. In fact, the authors only used 8 repetitions for each experimental condition. On top of that, the authors mention that outliers and errors were excluded. While mentioning that 91% of total trials were kept, it would be important to include a table with the number of trials, per condition, per participants, that were included in the calculation of the averages used in the analyses. The same thing for experiment 2, in which the number of repetition was augmented to 10, which is more acceptable, but still quite low. Given the little amount of data, it would be appropriate to perform the statistical analyses on medians, instead of means, which are less susceptible to extreme values.*

**Response**: Thank you for your comment. Following this suggestion, we have taken two actions in the current manuscript. First, the numbers of valid trials in each condition and participant are shown in Supplemental Tables 1 and 2. Second, as in the reply to comment #1.5, we conducted two-way repeated-measures ANOVAs on the means of log-transformed response times. Based on this change, we have added sentences as follows: “As a result, data obtained from 91.320% of the total trials were analyzed (see Table S1 for the number of valid trials in each condition). Moreover, the repetitions of trials in each condition were smaller than those in previous studies [7,18], which can bias the distribution of RTs. To adjust the distribution, we conducted statistical significance tests on the means of log-transformed RTs as in several previous studies that analyzed RTs [40,41].” (pp. 13–14, lines 204–209); “The number of valid trials in each condition is shown in Table S2.” (p. 18, line 284).

**Table S1.**

The numbers of valid trials in each condition and participants in Experiment 1.

| ID | Go/no go | | Joint | |
| --- | --- | --- | --- | --- |
|  | Comp | Incomp | Comp | Incomp |
| #1 | Excluded because of system trouble | | | |
| #2 | Excluded because of system trouble | | | |
| #3 | 6 | 7 | 8 | 8 |
| #4 | 8 | 8 | 7 | 7 |
| #5 | 7 | 8 | 8 | 6 |
| #6 | 8 | 6 | 7 | 8 |
| #7 | 8 | 8 | 8 | 7 |
| #8 | 8 | 8 | 8 | 8 |
| #9 | 6 | 7 | 6 | 7 |
| #10 | 7 | 8 | 7 | 8 |
| #11 | 7 | 8 | 8 | 6 |
| #12 | 7 | 8 | 8 | 8 |
| #13 | 7 | 8 | 8 | 7 |
| #14 | 8 | 7 | 7 | 8 |
| #15 | 5 | 8 | 7 | 8 |
| #16 | 3 | 5 | 4 | 8 |
| #17 | 6 | 8 | 8 | 8 |
| #18 | 8 | 8 | 8 | 5 |
| #19 | 8 | 8 | 7 | 8 |
| #20 | 8 | 8 | 8 | 8 |

Note. Error and outlier trials were excluded from the valid trials.

**Table S2.**

The numbers of valid trials in each condition and participants in Experiment 2.

| ID | Go/no go | | Joint | | Partner gaze | | Own gaze | | Both gaze | |
| --- | --- | --- | --- | --- | --- | --- | --- | --- | --- | --- |
|  | Comp | Incomp | Comp | Incomp | Comp | Incomp | Comp | Incomp | Comp | Incomp |
| #1 | 10 | 9 | 10 | 10 | 10 | 10 | 10 | 10 | 10 | 8 |
| #2 | 10 | 10 | 9 | 10 | 10 | 9 | 10 | 9 | 9 | 9 |
| #3 | 10 | 10 | 10 | 10 | 10 | 10 | 9 | 10 | 10 | 10 |
| #4 | 10 | 10 | 10 | 9 | 10 | 10 | 10 | 10 | 10 | 10 |
| #5 | 10 | 9 | 8 | 9 | 8 | 10 | 10 | 9 | 9 | 10 |
| #6 | 8 | 7 | 10 | 10 | 10 | 10 | 9 | 10 | 10 | 9 |
| #7 | 9 | 9 | 9 | 10 | 10 | 10 | 10 | 9 | 9 | 10 |
| #8 | 10 | 10 | 10 | 8 | 10 | 10 | 10 | 10 | 9 | 10 |
| #9 | 7 | 10 | 8 | 10 | 10 | 9 | 10 | 10 | 9 | 10 |
| #10 | 10 | 10 | 9 | 8 | 9 | 10 | 10 | 10 | 9 | 9 |
| #11 | 8 | 8 | 9 | 10 | 10 | 10 | 10 | 10 | 10 | 8 |
| #12 | 10 | 9 | 10 | 10 | 10 | 10 | 10 | 9 | 10 | 10 |
| #13 | 10 | 9 | 10 | 10 | 10 | 10 | 10 | 10 | 10 | 9 |
| #14 | 9 | 9 | 10 | 10 | 10 | 9 | 10 | 10 | 9 | 9 |
| #15 | 9 | 9 | 10 | 10 | 10 | 10 | 10 | 10 | 10 | 10 |
| #16 | 10 | 8 | 10 | 10 | 9 | 8 | 10 | 10 | 10 | 10 |
| #17 | 10 | 10 | 9 | 9 | 10 | 10 | 10 | 9 | 10 | 9 |
| #18 | 9 | 10 | 10 | 10 | 10 | 10 | 9 | 10 | 10 | 9 |
| #19 | 10 | 9 | 10 | 10 | 9 | 10 | 10 | 10 | 10 | 9 |
| #20 | 10 | 9 | 10 | 10 | 9 | 9 | 10 | 9 | 10 | 10 |
| #21 | 8 | 9 | 10 | 10 | 8 | 10 | 9 | 10 | 10 | 9 |
| #22 | 10 | 10 | 10 | 9 | 9 | 10 | 10 | 10 | 9 | 10 |
| #23 | 8 | 7 | 10 | 10 | 10 | 10 | 9 | 10 | 10 | 9 |
| #24 | 9 | 9 | 10 | 9 | 10 | 9 | 9 | 10 | 10 | 10 |
| #25 | 10 | 10 | 9 | 10 | 10 | 10 | 10 | 10 | 10 | 10 |
| #26 | 10 | 10 | 10 | 10 | 10 | 9 | 9 | 10 | 10 | 9 |
| #27 | 10 | 10 | 8 | 10 | 9 | 10 | 10 | 10 | 9 | 9 |
| #28 | 10 | 9 | 10 | 9 | 10 | 10 | 10 | 8 | 10 | 10 |
| #29 | 10 | 10 | 9 | 10 | 10 | 10 | 9 | 9 | 10 | 10 |
| #30 | 10 | 9 | 10 | 10 | 10 | 9 | 8 | 10 | 10 | 9 |
| #31 | 10 | 9 | 10 | 10 | 10 | 9 | 9 | 10 | 10 | 10 |
| #32 | 10 | 10 | 9 | 9 | 10 | 10 | 10 | 10 | 9 | 9 |
| #33 | 10 | 10 | 10 | 10 | 10 | 9 | 10 | 9 | 10 | 10 |
| #34 | 10 | 10 | 10 | 9 | 9 | 10 | 9 | 10 | 10 | 8 |
| #35 | 10 | 10 | 8 | 8 | 10 | 10 | 9 | 10 | 10 | 10 |
| #36 | 10 | 10 | 10 | 9 | 10 | 10 | 9 | 10 | 10 | 10 |
| #37 | 8 | 10 | 10 | 10 | 10 | 9 | 10 | 10 | 10 | 10 |
| #38 | 9 | 7 | 10 | 10 | 9 | 10 | 10 | 10 | 10 | 10 |
| #39 | 10 | 9 | 10 | 10 | 9 | 10 | 10 | 10 | 10 | 10 |
| #40 | 10 | 10 | 9 | 10 | 10 | 10 | 10 | 10 | 10 | 10 |
| #41 | 10 | 10 | 10 | 9 | 10 | 9 | 10 | 10 | 10 | 10 |
| #42 | 9 | 10 | 10 | 10 | 10 | 10 | 9 | 9 | 9 | 6 |
| #43 | 10 | 10 | 9 | 10 | 10 | 10 | 10 | 10 | 10 | 10 |
| #44 | 10 | 6 | 10 | 10 | 10 | 10 | 9 | 9 | 10 | 10 |

Note. Error and outlier trials were excluded from the valid trials.

**Comment #1.9**

*An important difference in the average RTs is present, when comparing results of experiments 1 and 2. The authors do not comment on this. Moreover, the amplitude of the simon effect in experiment 1 is virtually non-existent. The authors do not comment on this.*

**Response**: Following this comment, we have added a paragraph in the General Discussion section (p. 22, lines 348–355): “The mean RTs were approximately 200 ms faster in Experiment 2 than in Experiment 1. This would stem from the difference in instructions between the two experiments. The participants were asked to give priority to accuracy followed by speed in Experiment 1 but to quickness followed by accuracy. The reason for the instruction of Experiment 1 was that correct responses were required for analyzing correct RTs. Similarly, the amplitudes of the joint Simon effect were larger in Experiment 2 than in Experiment 1 (Fig 2b vs. 3b), which may stem from the difference in instructions. This would be because fast responses are less likely to contain noise and artifacts.”

**Comment #1.10**

*While consistently finding a joint simon effect on both experiment (although the amplitude of this effect greatly differs across the two), the authors did not find a gaze modulation of this effect. The authors proceeded in interpreting this null effect as evidence that i) either visualizing gaze information does not convey partner’s intentions or ii) gaze visualization does not affect the joint simon effect. The authors correctly mention that visualizing gaze direction on a mirror placed in front of participants might have hampered the effects, as well as the fact that a control condition without the partner’s avatar was not included. However, these limitations are not the only possible reasons for this null interaction effect. In fact, the avatars adopted in the study were very basic. It is important for the generation on the joint simon effect that participant feel the presence of the partner and interpret the avatar as conveying humans’ intentions. It is unclear whether participants interpreted the avatar’s choices as coming from a human or not. The latter option could explain the lack of gaze visualization effect.*

**Response**: We understand that the comment expresses concern that interpreting the avatar’s action as coming from a nonhuman may influence the present results. Our participants understood that the partner avatar was controlled by a partner because they had been instructed before the joint task. However, this instruction was not noted in the previous draft; thus, we have added a sentence to explain this. The manuscript reads as follows (p. 12, lines 186–188): “The participants received either a red or green color and were instructed to perform the joint task with their own avatar and another avatar that was controlled by a partner.”

**Comment #1.11**

*Moreover, the lateralized placement of the display could greatly affect results. Each participant should be tested twice, in order to control display’s placement on the amplitude of the simon effect. At the least, this factor should be counterbalanced across participants and its effect on the compatibility effect should be tested before any other analyses. It is also not mentioned whether participant were right-handed and which hand was used to deliver responses. The combination of all these factors could account for a simon effect size variability.*

**Response**: Thank you for your valuable comment. We agree that at least the counterbalance of lateral positions was required. We did counter the lateral positions across participants, but did not note that in the previous manuscript. We also agree with the comment that the details of participants’ handedness should be noted. Therefore, we have added descriptions as follows: “The lateral positions were counterbalanced across the participants.” (p. 11, lines 165–166); “Sixteen participants were right-handed, 2 were left-handed, and 2 were ambidextrous.” (p. 9, lines 130–131).” (b2) “Thirty-eight participants were right-handed, 5 were left-handed, and 1 was ambidextrous.” (p. 16, lines 253–254).

**Comment #1.12**

*As a general remark, English writing is quite bad. Even important words used multiple times in the article such as “square” for the target shape, are consistently misspelled. Revision with a native speaker is suggested for future submissions.*

**Response**: We apologize for the typos in the previous version. The current draft has been proofread and corrected by native speakers.

**Reviewer #2:**

**Comment #2.1**

*Firstly, starting with the abstract it is not clear what the authors refer at when using the term “intention”. This becomes even more ambiguous in the introduction, as they state on line 62: “… intention of a partner, a belief that a partner perceives stimuli”. However, this is more similar to the definition of Theory of mind and not with that of intentionality or perceiving mental states in a partner. Later on, they review the study by Stenzel et al, in which the authors however manipulated intentionality attribution towards the robot not the perception of TOM (see Marchesi et al 2017 , for a discussion about how an agent can be perceived as intentional even without attributing to it a tom). Such theoretical lack in the definition of intention, is then reflected in the experimental design of experiment 2. Why showing the gaze of the participants themselves or of their partners should have modulated the JSE, if participants were always controlling remotely their avatar, and by task instruction they were obliged to act in a complementary manner, there was no willingness in their actions. On the same issue, later on (see line91), the authors state: ”Given that one’s gaze convey mental states ….. visualizing a partner’s gaze would enhance the intentionality to the perceiver”. This is incorrect, visualizing partner gaze facilitates the decoding of their mental states but does not change whether we perceived them intentional or not-intentional agents. Moreover, the facilitation effect elicited by partner gaze is limited to when the gaze is directed toward a target/goal, otherwise it simply acts as a distracting stimulus that grabs participants attention from the task, or as an alerting one, which I think it may happen in experiment 2 (see below).*

**Response**: Thank you for your suggestive comment. We agree with the comment that our definition of “intention” is more similar to that of the theory of mind or mentalizing. We acknowledge the confusion in the definition. Therefore, we have revised the manuscript entirely to clarify our logic. In particular, we hypothesized that visualizing a partner’s gaze would enhance the joint Simon effect because it is an important cue to mentalize others’ internal states.

For the abstract, the manuscript now reads (pp. 2–3, lines 18–36): “… This effect has been considered to be influenced by the social factors of a partner: sharing stimulus-action representation. According to this account, virtual interactions through their avatars would produce the joint Simon effect even when the partner did not physically exist in the same space because the avatars are intentional agents… We hypothesized that visualizing the partner’s gaze would enhance the joint Simon effect because gaze is a cue to mentalize others’ internal states. The results showed that avatar-based interactions more significantly delayed responses for the incompatible than for the compatible stimulus. However, inconsistent with our hypothesis, visualizing the partner’s gaze position did not influence responses for spatially competing stimuli. These results suggest that virtual interactions through avatar agents can produce the joint Simon effect even when the partner does not physically exist in the same space.”

For the introduction section, the phrase “a belief that a partner perceives stimuli, evaluates them, and decides subsequent actions autonomously as a human” was eliminated to avoid confusions.

The sixth paragraph in the Introduction section was entirely revised to clarify our logics (pp. 7–8, lines 100–113): “This study also focuses on whether the visualization of a partner’s gaze can improve the joint Simon effect because the gaze is an important cue to mentalize others’ internal states. Since one’s eyes can convey mental states such as emotion, interest, and thought [28,29], the gaze direction captures attention (joint attention: [30]). The joint attention has been observed for not only the gaze of humans but also that of robots or humanoids [31,32]. Several studies have shown that visualizing a partner’s gaze promotes effective interactions in joint task situations [33,34]. Brennan et al. [35] conducted a joint visual search task in which a pair of participants was asked to collaboratively search for a target from distractors. When the attended location of a partner was visualized, participants adjusted their search to avoid the area being attended by the partner, resulting in a decreased overlap of search patterns with the partner. Gaze visualization has been reported to facilitate social interactions even when the gaze is produced by avatars [36]. Therefore, we hypothesized that the visualizing partner’s gaze can enhance the joint Simon effect because the visualization is a cue to read a partner’s internal states.”

A sentence in the last paragraph of the Introduction section were revised to clarify our hypothesis (p. 9, lines 123–124): “Given the co-representation account, the visualization of a partner would enhance the joint Simon effect by promoting mentalizing the partner’s internal states.”

Based on the above changes, relevant sentences in the Discussion section were revised as follows (p. 21, lines 328–339): “One possibility is that visualizing a partner’s gaze position may not promote mentalization of the partner’s internal states. In daily situations, persons detect another person’s eyes [29,30], recognize the direction of their eyes, and attend to an object that the other person is looking at [42]. In contrast, in Experiment 2, where the partner’s gaze position was visualized as a marker on a mirror, which is less common in daily situations. In particular, the detection of the eyes was not required due to visualization methods, which may reduce mentalization of the partner’s internal states. Nonetheless, previous studies have shown that visualizing a partner’s gaze improves joint task performance, especially for avoiding the duplication of efforts between two persons [33–35]. This duplication avoidance requires one’s own behaviors to be adjusted by mind reading. Therefore, manipulating gaze visualization would promote mentalization of the partner’s internal states.”

Finally, relevant sentences in the conclusion were revised as follows (p. 23, lines 369–373): “To summarize, our findings revealed that (a) virtual interactions through avatar agents had an effect on the joint Simon effect in an immersive virtual environment, whereas (b) the partner’s gaze visualization did not. These results suggest that virtual interactions promote sharing the stimulus-action representation even when the partner does not physically exist.”

**Comment #2.2**

*Second the introduction lacks in a proper revision of the literature. First, beside the action co-representation account and the referential coding one, there are other accounts. For instance, other accounts started to underline the role of spatial response coding processes (e.g., Guagnano, Rusconi, & Umiltà, 2010; Dolk et al., 2011; Dittrich, Rothe, & Klauer, 2012; Lugli, Iani, Milanese, Sebanz, & Rubichi, 2015; Dittrich, Bossert, Rothe-Wulf, & Klauer, 2017; Dittrich, Pufe, & Klauer, 2017; Michel, Bölte, & Liepelt, 2018; see also see Dolk et al., 2014 and Prinz, 2015 for reviews). In addition, when reviewing literature about the JSE and robots the authors should consider also the work by Ciardo et al 2022, showing that in a JSt ask with a humanoid robot the JSE emerge irrespectively of whether the robot was remotely controlled by the human-partner of by a computer program; and the study by Sahaï et al 2019, showing that the JSE emerges only for humanoid robots but not for servomotor robots.*

**Response**: Following this constructive comment, we cited (a) articles focusing on the role of spatial response coding processes and (b) Ciarde et al. (2022).

For the former, the manuscript reads (p. 6, lines 81–85): “Another account has focused on the role of the spatial response coding process [15,16]. In this account, it is presumed that external stimulus events and internal response events are represented as common formats and that responses are facilitated when the stimulus and response events are similar [17].”

For the latter, the manuscript reads (pp. 5–6, lines 71–76): “A recent study found that joint action with a humanoid robot produced the joint Simon effect [11]. This effect was produced even when the robot was controlled by an algorithm, which is partially inconsistent with the results from Stenzel et al. [8]. The incongruency may be because the humanoid used in Ciardo et al. [11] is more human in appearance than that used in Stenzel et al. [8].”

**Comment #2.3**

*Third, the results of the two experiments showed that a SE emerged in the go/no-go condition. I see that such effect does not differ from zero, however, as indicated by the lack of significant interaction it does not differ neither from the SE elicited in the Joint conditions of both experiments. The authors completely missed to address such result in their discussion.*

**Response**: We agree with the comment that the ANOVA results show a non-significant difference in the Simon effect between the go/no go and joint tasks. However, the results of the t-test showed that the Simon effect was significantly larger than 0 for the joint conditions but not for the go/no go condition. Therefore, we added a limitation for this issue in the general discussion as follows (p. 22, lines 356–360): “A limitation is that we did not obtain the two-way interaction between the compatibility and task because the Simon effect was slightly produced for the go/no go task. Instead, we analyzed whether the strength of the Simon effect (Fig 2b and 3b) was larger than 0. Therefore, the present results show the Simon effect for the joint conditions but do not necessarily show the difference between the go/no go and joint conditions.”

Moreover, we eliminated discussions for null effect of gaze visualization from the conclusion.

**Comment #2.4**

*On line 110 the authors should better address why the referential coding predicts a lack of JSE when the gaze is displayed.*

**Response**: We have revised the relevant sentence as follows (p. 9, lines 124–126): “However, the referential coding account predicts that gaze visualization does not affect the joint Simon effect because the gaze has few cues for the spatial position of participants.”

**Comment #2.5**

*Line108 the authors should explicitly say that the avatars were always remotely controlled, also in Experiment 1*

**Response**: We added a sentence as follows (p. 8, lines 115–117): “In the two experiments, participants always remotely controlled their avatars using three-point tracking (head, left hand, and right hand).”

**Comment #2.6**

*Where participants only right-handed? If not, please specify how many left-handed participants took part in both Experiment 1 and 2*

**Response**: As noted in the response to comment #1.11, we have added the details of participants’ dominant hand in Experiments 1 and 2: “Sixteen participants were right-handed, 2 were left-handed, and 2 were ambidextrous.” (p. 9, lines 130–131); “Thirty-eight participants were right-handed, 5 were left-handed, and 1 was ambidextrous.” (p. 16, lines 253–254).

**Comment #2.7**

*For replicability it would be better to have a figure or a video displaying the environment used in the syudy*

**Response**: Following this comment, we uploaded Supporting Videos 1 and 2. Video S1 shows the trial sequence of joint condition in Experiments 1 and 2. Video S2 shows the trial sequence of both gaze condition in Experiment 2.

**Comment #2.8**

*Which was the maximum time allowed for responses? The JSE, as any spatial compatibility effect emerges from the exogenous orienting of attention, and thus it tends to decrease in magnitude for long RTs*

**Response**: The maximum time was 2 seconds. To explain this, we have revised a relevant sentence and Figure 1. The revised sentence now reads (p. 12, lines 177–178): “Subsequently, a target square was presented at the left or right side of the display until a response or 2 seconds elapsed.”

**Comment #2.9**

*Line 169: How long last the blank screen between trials?*

**Response**: The blank screen between trials lasted 1.5 seconds. Because the details of the blank duration in the previous draft were inaccurate, we have revised those as follows (p. 12, line 182): “After a 1.5-second blank, the next trials began.”

**Comment #2.10**

*Line 177: It would be better to remove from the analysis also the other member of the pair given that the task relies on complementarity*

**Response**: Following this comment, we removed the data obtained from the pair of participants and re-analyzed all data. The manuscript now reads (p. 13, lines 200–201): “Data obtained from one pair of participants were entirely excluded from the analyses due to system troubles that prevented the recording of RTs.” Based on the changes, we updated statistical values in the Results and Discussion section.

**Comment #2.11**

*Line 188: RTs are a measure of the time interval between two events (stimulus and response), they do not measure size, so they cannot be “greater than”, but they can be longer or shorter.*

**Response**: Following this comment, we replaced the word “greater” with “longer” in the relevant sentences.

**Comment #2.12**

*When reporting the results it will be better to also report means of each experimental condition instead that simply report a plot. If someone wants to estimate the effect size, it will be impossible to calculate it without means and sd values.*

**Response**: Following this comment, we reported the values of means and SDs.

For Fig 2a, the manuscript reads as follows (p. 14, lines 212–215): “For the go/no go task, the mean RTs (SDs) were 0.799 (0.208) in the compatible condition and 0.809 (0.209) in the incompatible condition. For the joint task, the mean RTs (SDs) were 0.805 (0.203) in the compatible condition and 0.821 (0.189) in the incompatible condition.”

For Fig 2b, the manuscript reads as follows (p. 15, lines 227–228): “The mean Simon effects (SDs) were 0.00222 (0.00705) for the go/no go task and 0.00363 (0.00530) for the joint task.”

For Fig 3a, the means and SDs are shown in Table S3.

**Table S3.**

The means and SDs for each condition in Experiment 2.

| Condition | Compatibility | Mean | SD |
| --- | --- | --- | --- |
| Go/no go | Comp | 0.591 | 0.096 |
|  | Incomp | 0.594 | 0.089 |
| Joint | Comp | 0.577 | 0.082 |
|  | Incomp | 0.592 | 0.085 |
| Partner gaze | Comp | 0.573 | 0.087 |
|  | Incomp | 0.590 | 0.090 |
| Own gaze | Comp | 0.587 | 0.085 |
|  | Incomp | 0.601 | 0.086 |
| Both gaze | Comp | 0.589 | 0.086 |
|  | Incomp | 0.604 | 0.092 |

For Fig 3b, the manuscript reads (p. 19, lines 294–297): “As in Experiment 1, we calculated the Simon effect (Fig 3b). The mean Simon effects (SDs) were 0.00138 (0.00899) for the go/no go, 0.00412 (0.00729) for the joint, 0.00478 (0.00696) for the partner gaze, 0.00369 (0.00719) for the own gaze, and 0.00433 (0.00887) for the both-gaze conditions.”

**Comment #2.13**

*I suggest the authors to put more effort in revising the English writing.*

**Response**: We apologize for the typos in the previous manuscript. As in the reply to Comment 1.12, the current draft was proofread and corrected by native speakers.
