# Supplementary material for: Effect of virtual interactions through avatar agents on the joint Simon effect
Source: PLoS One. 2025 Jan 10;20(1):e0317091. doi: 10.1371/journal.pone.0317091 (PMC11723602; doi:10.1371/journal.pone.0317091)
Supplement: Attachment — Submitted filename: PESCI_review_PlosOne24.docx [file pone.0317091.s007.docx]

**Effect of virtual interactions through avatar agents on the joint Simon Effect** – *Plos One* 2024

The authors present two studies aiming at investigating the joint Simon Effect in a virtual environment. Specifically, they aim at investigating the effect of the interaction between users’ avatars on it, in immersive virtual environments. Participants remotely controlled one avatars each. In Experiment 1, they performed a Go/NoGo task and a Joint Simon task. Avatars were always controlled from a first-person perspective. In Experiment 2, they performed the same tasks, but also visualizing the other participant’s gaze. Regarding Exp. 1, the authors find no difference in the effect of compatible vs incompatible stimuli between the Go/NoGo and the Joint Simon task when looking at the RT. When they quantify the Simon effect separately for the two tasks using a measure of difference between compatible and incompatible, they find a significant difference between tasks, with a Simon effect only in the Join Simon task but not it the Go/NoGo one. In experiment 2, they seem to replicate the results from Exp 1, while also reporting a null effect of the presence of visual cues regarding a virtual partner’s gaze.

Although the set-up and the experimental rationale sound new and potentially suitable to answer an open question in the literature, I find quite a few technical, analytical and interpretational points of concern. Apart from the overall poor grammatical and lexical choices, all the descriptions in the methods section need a thorough revision and broadening.

Starting from line 137, it is never clear to me whether the two participants for each dyad were in the same physical room (divided in two areas), or in two separate ones. Fig 1a does not help on this.

Moreover, at line 163 the authors describe shortly the presence of a mirror in the virtual scene, but it is never specified what the participant saw reflected on it in the various conditions, in both experiments. This is crucial to understand and to be precisely described, since it is where the gaze of the partner is later visualized.

Line 173, the authors describe a “touch” subjects were supposed to perform in the virtual environment, of which the RT were recorded. Again, there are no specifications on the instruments used to record these, if the subjects actually moved or they “only” used to joystick, etc. Since this is the main variable of the study, I strongly suggest a better description of all the technical related to it.

Line 185, it is, in my opinion, crucial to specifically explain the similarities and differences between instructions (never explicitly stated through the whole manuscript) between the Go/NoGo and the Joint Simon task. Since the (rather small but consistent) results obtained might be due also to attentional/visual cues differently distributed in the environment, I find this point to be essential.

Line 191, it is not clear to me how the participants knew the goal of the partner. I understand they saw the “gaze” of the other, but how could they draw whether that was actually their target/goal?

Line 204, were the outliers selected for each condition separately? It does not sound so, and I would recommend to find them separately for each condition at the lowest level of the factorial design.

Regarding the interpretation of the results from exp 2, it looks hard to disentangle attentional processes driven by visual, fundamental cues (the two dots on the mirror) from more complex attentional processes related to social processing. In order to clearly discuss social factors, I would have found a stronger argument if the same effect was present with the dots over-imposed on a full face. What is the difference between two dots and an arrow otherwise?

Line 264/276, the conditions in exp 2 absolutely need a better explanation. I do not understand what Go/NoGo and Joint Simon are if not the same than in exp. 1, but the authors explain that exp 2 was performed with the presence of the other’s and one’ own gaze in all conditions. I understand my view is confused, and I believe my confusion reflects the poor description of all these details in the manuscript.

Further methodological and general major points: the authors cite relevant articles in the field, some of them applying the same paradigm in non-virtual scenarios, thus I would expect they ran a power analysis to fully justify their samples. Lastly, a line 155 the figure of the avatars and the virtual environment would really help a lot. As shown in the majority of works in the literature of immersive virtual reality, rather than suggesting the reader to watch the supplementary videos, full screenshots of the virtual agents and environment should be shown. Generally, I find the graphic choices for the plots and experimental set-up representation poor.
