# Supplementary material for: Effect of virtual interactions through avatar agents on the joint Simon effect
Source: PLoS One. 2025 Jan 10;20(1):e0317091. doi: 10.1371/journal.pone.0317091 (PMC11723602; doi:10.1371/journal.pone.0317091)
Supplement: Attachment — Submitted filename: Response to Reviewers.docx [file pone.0317091.s008.docx]

Response to the review of “Effect of Virtual Interactions Through Avatar Agents on the Joint Simon Effect” by Y. Harada, T. Arima, & M. Okada. Comments provided by Reviewer #3 (in italics) and our specific responses to them are shown below. Our responses include page and line numbers indicating the revisions made to the manuscript.

**Reviewer #3**

*The authors present two studies aiming at investigating the joint Simon Effect in a virtual environment. Specifically, they aim at investigating the effect of the interaction between users’ avatars on it, in immersive virtual environments. Participants remotely controlled one avatars each. In Experiment 1, they performed a Go/NoGo task and a Joint Simon task. Avatars were always controlled from a first-person perspective. In Experiment 2, they performed the same tasks, but also visualizing the other participant’s gaze. Regarding Exp. 1, the authors find no difference in the effect of compatible vs incompatible stimuli between the Go/NoGo and the Joint Simon task when looking at the RT. When they quantify the Simon effect separately for the two tasks using a measure of difference between compatible and incompatible, they find a significant difference between tasks, with a Simon effect only in the Join Simon task but not it the Go/NoGo one. In experiment 2, they seem to replicate the results from Exp 1, while also reporting a null effect of the presence of visual cues regarding a virtual partner’s gaze.*

*Although the set-up and the experimental rationale sound new and potentially suitable to answer an open question in the literature, I find quite a few technical, analytical and interpretational points of concern. Apart from the overall poor grammatical and lexical choices, all the descriptions in the methods section need a thorough revision and broadening.*

**Response**: Thank you very much for your helpful review of the manuscript. Based on the comments and suggestions, we have made some changes to the manuscript. Specifically, we entirely revised the method sections in two experiments to clarify the details of experimental settings and procedures.

***Comment #1:*** *Starting from line 137, it is never clear to me whether the two participants for each dyad were in the same physical room (divided in two areas), or in two separate ones. Fig 1a does not help on this.*

**Response**: Two participants and VR sets were separated by different experiment rooms. To clarify this, we revised the sentences as follows (p. 10, lines 142-143): “A virtual reality (VR) system was installed in separate rooms (Fig 1a).” We also revised Fig 1a to clarify that two participants were in separate rooms.

**Fig 1. Illustration of experimental settings and virtual environments.** (a) A pair of VR systems. Each system comprised an HMD, two controllers, two sensors, and a PC. A local network connected the systems. (b) Virtual environments in Experiment 1. (c) Virtual environments in Experiment 2. The environments comprised participants’ avatars, a mirror, a display for targets, buttons, and gaze markers (only in Experiment 2).

***Comment #2:*** *Moreover, at line 163 the authors describe shortly the presence of a mirror in the virtual scene, but it is never specified what the participant saw reflected on it in the various conditions, in both experiments. This is crucial to understand and to be precisely described, since it is where the gaze of the partner is later visualized.*

**Response**: To address what the mirror reflected, we explained the details of virtual environments in the two experiments.

The added sentences in Experiment 1 read (pp. 11-12, lines 164-175): “Fig 1b shows the schematic illustration of virtual environments in Experiment 1. The virtual environments comprised participants’ avatars, a mirror, and a display for target presentation. Because the position coordinates of the head and both hands corresponded to those of HMD and two controllers, participants could move the avatars as if they were moving their bodies. One avatar existed in the go/no-go task, while two avatars (one is own, the other one is a partner) existed in the joint Simon task. The mirror reflected the avatars of participants, aiming to help participants perceive the status of their avatars. The display for targets was presented between the mirror and the button, where a red or green square was presented on the left or right side. The buttons were located at the front of the participants’ avatar and could detect contact with the avatar’s hand using the collision enter function in Unity. Participants were asked to push their avatars’ hand into the button named “red” when their target was red or that named “green” when their target was green.”

The added sentences in Experiment 2 read (p. 19, lines 299-307): “Fig 1c shows the schematic illustration of virtual environments in Experiment 2. The first-person avatars, mirror, and buttons were identical to those of Experiment 1. The gaze positions were shown as a black or white marker on the mirror in the immersive virtual environments. The gaze markers were presented in the own-gaze, partner-gaze, and both-gaze conditions but not presented in the go/no-go and joint Simon conditions. The gaze marker of own-gaze position was presented in the own-gaze condition, while that of partner-gaze position was presented in the partner-gaze condition. The gaze markers of two participants were presented in both-gaze conditions (their markers could be distinguished due to colors).”

We also revised Fig 1b and c to clarify the details of virtual environments in the two experiments. Revised Fig 1 is shown in the response to Comment #1.

***Comment #3:*** *Line 173, the authors describe a “touch” subjects were supposed to perform in the virtual environment, of which the RT were recorded. Again, there are no specifications on the instruments used to record these, if the subjects actually moved or they “only” used to joystick, etc. Since this is the main variable of the study, I strongly suggest a better description of all the technical related to it.*

**Response**: To explain the touch, we added sentences as follows (p. 11, lines 169-178): “Because the position coordinates of the head and both hands corresponded to those of HMD and two controllers, participants could move the avatars as if they were moving their bodies…. The buttons were located at the front of the participants’ avatar and could detect contact with the avatar’s hand using the collision enter function in Unity. Participants were asked to push their avatars’ hand into the button named “red” when their target was red or that named “green” when their target was green.”

***Comment #4:*** *Line 185, it is, in my opinion, crucial to specifically explain the similarities and differences between instructions (never explicitly stated through the whole manuscript) between the Go/NoGo and the Joint Simon task. Since the (rather small but consistent) results obtained might be due also to attentional/visual cues differently distributed in the environment, I find this point to be essential.*

**Response**: Following this suggestion, we revised the procedure section, especially to clarify the instructions for the two tasks.

The instructions for the current manuscript in Experiment 1 now read (pp. 11-12, lines 181-204): “The experiment was conducted in the order of go/no-go practice, go/no-go test, and joint Simon tasks. Before the practice, participants received a target color (red or green). Participants were asked to push their hand to a corresponding button when the target of the received color was presented, irrespective of target locations, but to ignore it when the non-target color was presented. After the practice (eight trials), the go/no-go task test was conducted. The trial sequence was as follows (Fig 2a). After a three-second count, a black fixation cross was presented at the center of the display for one second. Subsequently, a target square was presented at the left or right side of the display until a response, or 2 seconds elapsed. The participants’ task was to touch the corresponding button that was virtually set in front of them when the target color was presented…. After the go/no-go task, the participants conducted the joint Simon task. The participants were asked to touch a corresponding button when the target color was presented, as in the go/no-go task, but to wait until a partner’s avatar touched a corresponding button when a non-target color was presented. The participants knew the target color of a partner via this instruction. A trial sequence was shown in Fig 2b (see Supporting Video 1 for the movie of the trial sequence). Similar to the go/no-go task, after the three-second count and fixation cross, a target square was presented on the left or right side of the display. The participants were asked to press a corresponding button as quickly and accurately as possible when their target color was presented.”

The instructions for the current manuscript in Experiment 2 now read (pp. 19-20, lines 304-314): “In Experiment 2, participants were instructed to prioritize speed followed by accuracy. This is because requiring quick responses would sharpen the effect of gaze visualization on the RTs. After calibrating the eye-tracking system, participants performed the go/no-go task. Subsequently, they conducted the joint Simon tasks under four conditions manipulated within experimental blocks (see Supporting Video 2 for the movie of a trial). Participants were instructed to touch a corresponding button when the target color was presented but to wait until a partner’s avatar touched a corresponding button when the non-target color was presented. For the own-, partner-, and both-gaze conditions, it was also explained that their gaze positions, those of their own, that of the partner, or that of both participants, were visualized in the mirror while conducting this task.”

***Comment #5:*** *Line 191, it is not clear to me how the participants knew the goal of the partner. I understand they saw the “gaze” of the other, but how could they draw whether that was actually their target/goal?*

**Response**: Regarding Comment #4, we revised the procedure details to clarify the instruction (p. 13, lines 199-203): “After the go/no-go task, the participants conducted the joint Simon task. The participants were asked to touch a corresponding button when the target color was presented, as in the go/no-go task, but to wait until a partner’s avatar touched a corresponding button when a non-target color was presented. The participants knew the target color of a partner via this instruction.”

***Comment #6:*** *Line 204, were the outliers selected for each condition separately? It does not sound so, and I would recommend to find them separately for each condition at the lowest level of the factorial design.*

**Response**: We agree with you. However, in the present study, selecting the outlier criteria for each condition would be challenging because the number of repetitions was small. There were 8 repetitions in Experiment 1 [total number = 32: 2 response (go, no go) x 2 compatibility (compatible, incompatible) x 8 repetitions] and 10 repetitions in Experiment 2 [total number = 40: 2 response (go, no go) x 2 compatibility (compatible, incompatible) x 10 repetitions]. In this case, standard deviations become larger, resulting in leaving some outlier response times.

The main text should note the reason for calculating outliers from the data pooled across conditions. Therefore, we added the reason in the analyses section (p. 15, lines 231-237): “One may argue that the outliers should be selected for each condition. However, in the present study, selecting the outlier criteria for each condition would be difficult because the number of repetitions was relatively smaller than those in previous studies [7,18] (8 repetitions in Experiment 1). In this case, standard deviations become larger, resulting in leaving some outlier RTs. Therefore, we calculated outliers based on the data pooled across the conditions.”

***Comment #7:*** *Regarding the interpretation of the results from exp 2, it looks hard to disentangle attentional processes driven by visual, fundamental cues (the two dots on the mirror) from more complex attentional processes related to social processing. In order to clearly discuss social factors, I would have found a stronger argument if the same effect was present with the dots over-imposed on a full face. What is the difference between two dots and an arrow otherwise?*

**Response**: We agree with the suggestion that it is hard to disentangle attentional processes from social cognitive processing in our results. Since this topic would be important for clarifying the underlying mechanism of our results, we added a discussion about this (p. 26, lines 416-424). The paragraph reads: “It may be important to investigate the effect of gaze visualization on the joint Simon effect from the perspective of social cognitive processing. Gazes convey social cognitive information such as the intention, goal of behaviors, and emotional states [29] by directing gazes to the subject of interest. In joint contexts, gazes can frequently be directed to a partner, which would be a cue to communicate information about joint tasks between persons. Given this, social communications through gaze directions may promote stimulus-response representation. Unfortunately, the present study cannot answer the question because it did not manipulate the direction of gazes and just presented gaze positions as dots. This possibility should be investigated in future studies.”

***Comment #8:*** *Line 264/276, the conditions in exp 2 absolutely need a better explanation. I do not understand what Go/NoGo and Joint Simon are if not the same than in exp. 1, but the authors explain that exp 2 was performed with the presence of the other’s and one’ own gaze in all conditions. I understand my view is confused, and I believe my confusion reflects the poor description of all these details in the manuscript.*

**Response**: We apologize for the confusion. The go/no-go and joint Simon tasks were the same between Experiments 1 and 2, but three joint conditions with visualized gaze positions were added in Experiment 2. To decrease the confusion, we revised the relevant sentences.

The preamble of Experiment 2 now reads (pp. 17-18, lines 273-278): “… The go/no-go and joint Simon conditions were identical to those in Experiment 1. In the other three conditions, participants performed the joint Simon task while their partner’s and/or their own gaze was visualized. In the partner-gaze condition, the gaze position of the partner was visible. In the own-gaze condition, the own gaze position was visible. In the both-gaze condition, gaze positions of both participants were visible.”

The Apparatus and materials section in Experiment 2 (pp. 19, lines 296-302) now reads: “The gaze positions were shown as a black or white marker on the mirror in the immersive virtual environments. The gaze markers were presented in the own-gaze, partner-gaze, and both-gaze conditions but not presented in the go/no-go and joint Simon conditions. The gaze marker of own-gaze position was presented in the own-gaze condition, while that of partner-gaze position was presented in the partner-gaze condition. The gaze markers of two participants were presented in both-gaze conditions (their markers could be distinguished due to colors).”

The procedure section in Experiment 2 now reads (pp. 19-20, lines 308-313): “… Participants were instructed to touch a corresponding button when the target color was presented but to wait until a partner’s avatar touched a corresponding button when the non-target color was presented. For the own-, partner-, and both-gaze conditions, it was also explained that their gaze positions, those of their own, that of the partner, or that of both participants, were visualized in the mirror while conducting this task. ...”

***Comment #9:*** *Further methodological and general major points: the authors cite relevant articles in the field, some of them applying the same paradigm in non-virtual scenarios, thus I would expect they ran a power analysis to fully justify their samples. Lastly, a line 155 the figure of the avatars and the virtual environment would really help a lot. As shown in the majority of works in the literature of immersive virtual reality, rather than suggesting the reader to watch the supplementary videos, full screenshots of the virtual agents and environment should be shown. Generally, I find the graphic choices for the plots and experimental set-up representation poor.*

**Response**: We originally determined the sample sizes based on previous works. We agree with you that the results of power tests would be useful to justify the sizes and detect statistically reliable effects. Following this suggestion, we added the results of the power tests to the Participants section.

The added sentences for Experiment 1 are as follows (pp. 9-10, lines 136-141): “The sample size was determined by previous studies investigating the joint Simon effect [6,9,18]. A priori power test was conducted with G*power, in which test family = “F tests,” statistical test = “ANOVA: Repeated measures, within factors,” effect size (*f*) = 0.31 (Experiment 2 of Dolk et al. [18]), alpha = .05, and power = 0.8 were entered. This test required 24 sample sizes to detect the joint Simon effect. The sample size of Experiment 1 was comparable to the required size.”

The added sentences for Experiment 2 are as follows (p. 18, lines 286-290): “A priori power test was conducted with G*power, in which test family = “F tests”, statistical test = “ANOVA: Repeated measures, within factors”, effect size (*f*) = 0.25, alpha = .05, and power = 0.8 were entered. This test required 34 sample sizes to detect statistically reliable effects. The sample size of Experiment 2 was larger than the required size.”

We also thank you for your comments about the graphics. As noted in the responses to Comments #1 and #2, we improved Figs 1 and 2 to clarify the details of experimental settings and stimulations.
